# Liquid crystal between two distributed Bragg reflectors enables multispectral small-pitch spatial light modulator

**DOI:** 10.1038/s41377-022-00882-w

**Published:** 2022-07-07

**Authors:** Junghyun Park, Kanghee Won, Young Kim

**Affiliations:** Advanced Sensor Lab, Device Research Center, Samsung Advanced Institute of Technology, Suwon, Republic of Korea

**Keywords:** Liquid crystals, Nanophotonics and plasmonics

## Abstract

The ability of controlling the phase of light at the subwavelength scale can be a game-changer due to its extraordinally wide angle-range in the wavefront shaping. By combining two conventional material and configuration, liquid crystal and distributed Bragg reflectors, we are getting close to this ultimate goal.

In 1801, Thomas Young demonstrated the wave behavior of light using the double-slit experiment, where waves passing through each slit interfered constructively or destructively forming the fringe pattern with bright and dark stripes. The key principle underlying associated with this phenomenon can be elucidated by the concept of the phase of light. The wave function at a certain point at a specific time is given by the superposition of waves propagating from the sources, and the bright spots are formed when the summation of each phase becomes an integer multiple of 2π.

The ability of manipulating the phase of light in each source by external control signals allows us to generate on-demand wavefront as desired. The spatial light modulator (SLM) is a gadget that enables such phase control at will and is composed of a one- or two-dimensional array of individual pixels that can change the amplitude or phase of reflected/transmitted light. Most conventional approaches for the SLMs rely on liquid crystal (LC) or micro electromechanical systems. We can find numerous applications based on SLMs including digital holographic systems, optical communication, and biomedical imaging, to name a few^1^.

Let us get back to the Young’s double-slit experiment. One can imagine that, as we decrease the gap between two slits, we observe the increased distance between the bright and dark stripes. The angle range between two consecutive bright or dark fringes is called the field of view, and plays a crucial role of important metrics because wider field of views allow enhanced performance in most applications, for example, larger eyebox in holography and increased sensing area in the light detection and ranging (LiDAR)^2^. Consequently, there has been considerable research on expanding the field of view by reducing the pixel size. Conventional LC-based SLMs, however, are subject to the limitation of reducing the pixel size. This is because they require enough vertical thickness, called cell gap, to achieve full 2π accumulated propagation phase. Thus the reduction of pixel sizes in horizontal dimension under a certain value may give rise to the fringing field of the electric field, which in turn causes deficient phase expression.

As alternative approaches, there has been considerable research toward the reduction of pixel sizes by invoking the reconfigurable metasurfaces^2–5^. Metasurfaces are arrays of optical scatterers with strong light-matter interaction that allow extremely localized optical response with a substantially suppressed crosstalk. By adding time-dependent variation of these responses from metasurfaces through active materials with tunable refractive indexes, one could implement novel SLMs with pixel sizes at the subwavelength regime. However, most metasurface-based SLMs to this day still suffer from limitations such as the narrow range of the phase change far below 2π and narrow bandwidth of the operating regime.

A recent research paper in Light: Science & Applications, entitled “High Resolution Multispectral Spatial Light Modulators based on Tunable Fabry-Perot Nanocavities”, by Kuznetsov’s group, introduces remarkable progress in seeking for the SLM solution with the small pixel, the wide phase-change range, and the multi-spectral response^6^. The configuration includes the LC encapsulated between the upper and lower distributed Bragg reflectors, forming a Fabry–Pérot cavity (Fig. 1). The incident beam from the upper side coupled into the resonator runs back and forth, and the over-coupled resonance occurs when the round trip phase becomes an integer multiple of 2π. The over-coupling dynamics allows 2π spectral phase as one sweep the wavelength. The large birefringence Δn = 0.29 of the nematic LC molecule, QYPDLC-001C, enables the experimental demonstration of near 2π under the applied bias V_rms_ of 8 V.

**Fig. 1:**
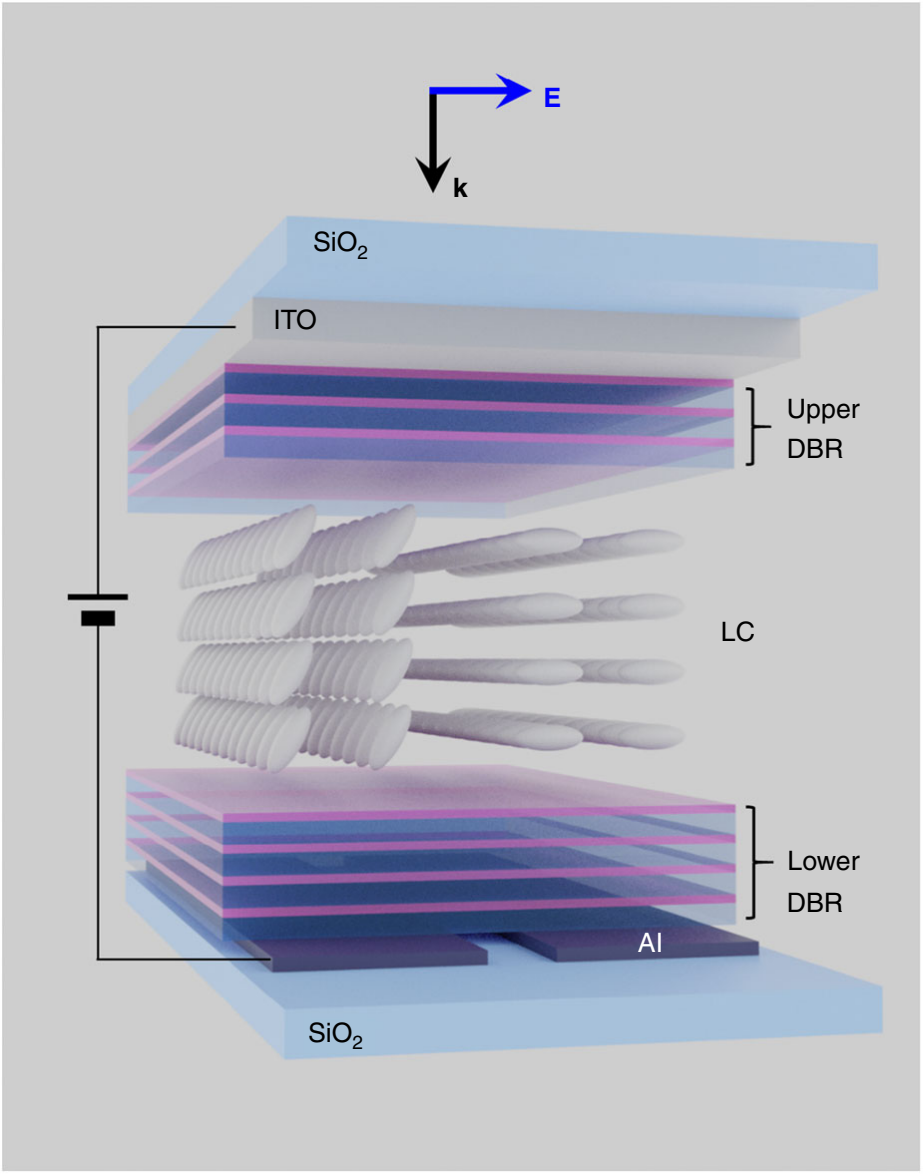
**Schematic view of the Fabry–Pérot cavity**. It is composed of the LC as a refractive-index changing material sandwiched between the upper and lower distributed Bragg reflectors, allowing for the reduced cell gap and the corresponding small pixel size for the wide field of view. The higher-order mode resonance in the cavity facilitates multi-spectral phase modulation in the visible regime

The proposed Fabry–Pérot-SLM features two distinguished points: the three simultaneous operating wavelengths in the visible regime (multispectral response) and the small pixel size of 1.14 µm. Indeed, those two factors are under trade-off relationship. The secret ingredient is a judiciously designed thickness of the cavity filled with LCs. If the authors used the fundamental mode in the Fabry–Pérot cavity, the required thickness for the fundamental Fabry–Pérot resonance would be given by the half wavelength, *t*_c_ = *λ*_0_/(4*n*), around 150 nm, where *t*_c_ is the cavity thickness, *λ*_0_ is the operating wavelength, and *n* is the refractive index of the LC. Such a thin cavity thickness would be advantageous for the small pixel size and the wide field of view, because it can suppress fringing field effects between neighboring pixels. Kuznetsov and his colleagues intentionally increased the cavity thickness to 530 nm (the original design of 750 nm), and they could employ higher order Fabry–Pérot resonances at the three wavelength regimes in the visible; red (*λ*_0_ of 640 nm) for 4^th^ order, orange (*λ*_0_ of 596 nm) for 5^th^ order, and blue (*λ*_0_ of 503 nm) for 6^th^ order.

Despite the increased cavity thickness 530 nm for the higher order modes, the total thickness of each pixel between the upper and lower electrodes is around ~2 µm, which is way smaller than that of conventional LC-based SLMs (~5 µm). This small thickness allowed the authors to achieve reduced pixel size down to 1.14 µm. They demonstrate multi-spectral programmable beam steering with field of view of ~18° as well as multi-spectral vary-focal lensing.

Despite the pioneering achievements in this work, there are still remaining tasks to be solved in the future. Although the phase response versus the applied bias in the non-pixelated structures show successful near-2π phase sweep, the beam steering in the real pixel-arrays results still exhibit non-vanishing side lobes. This kind of degradation in performance under the migration from non-pixelated unit cell characterization to pixelated array operation is observed quite often even in state-of-the-art studies, but should be resolved in the real applications. This may be ascribed to the potential crosstalk between 1.14-μm-pitch pixels. If we define the pixel size *not by* its physical appearance (the pitch of electrodes) *but by* its functioning unit, i.e., the pitch of pixels that allow 2-pixel supercell showing the side mode suppression ratio more than 10 dB, for example, the claimed smallest pixel of 1.14 µm could be slightly increased. To be used in the real-life applications, further efforts need to be made to suppress the undesirable side lobes. In addition, the response time or the switching speed, which was not comprehensively studied in this work, may also be addressed. Nevertheless, the proposed platform of LCs in two distributed Bragg reflectors is a significant contribution to the small-pixel SLMs with multi-spectral response and could be extended to two-dimensional arrays or transmissive type in the future (Table 1).

**Table 1:** Channel/pixel size of LC-based SLMs

Dimension	Type	
	Reflective	Transmissive
One-dimensional	1.14 µm (this work)	1.14 µm ^3^
	1.60 µm ^7^	2 µm ^8^
Two-dimensional	3.74 × 3.74 µm^2^ ^9^	36 × 36 µm^2^ ^11^
	1 × 9 µm^2^ ^10^	
